# Physicochemical, Spectroscopic, and Chromatographic Analyses in Combination with Chemometrics for the Discrimination of the Geographical Origin of Greek Graviera Cheeses

**DOI:** 10.3390/molecules25153507

**Published:** 2020-07-31

**Authors:** Kornilia A. Vatavali, Ioanna S. Kosma, Artemis P. Louppis, Anastasia V. Badeka, Michael G. Kontominas

**Affiliations:** 1 Laboratory of Food Chemistry, Department of Chemistry, University of Ioannina, 45110 Ioannina, Greece; kvatavali26@gmail.com (K.A.V.); i.kosma@uoi.gr (I.S.K.); 2 CP FoodLab Ltd., 25 Polyfonti Str. P.O. Box: 28729, Strovolos- Nicosia 2082, Cyprus; artemislouppis@gmail.com

**Keywords:** geographical discrimination, graviera cheese, physicochemical parameters, fatty acids, volatile compounds, minerals, chemometric analysis

## Abstract

Seventy-eight graviera cheese samples produced in five different regions of Greece were characterized and discriminated according to geographical origin. For the above purpose, pH, titratable acidity (TA), NaCl, proteins, fat on a dry weight basis, ash, fatty acid composition, volatile compounds, and minerals were determined. Both multivariate analysis of variance (MANOVA) and linear discriminant analysis (LDA) were applied to experimental data to achieve sample geographical discrimination. The results showed that the combination of fatty acid composition plus minerals provided a correct classification rate of 89.7%. The value for the combination of fatty acid compositions plus conventional quality parameters was 94.9% and for the combination of minerals plus conventional quality parameters was 97.4%. When cheeses of the above five geographical origins were combined with previously studied graviera cheeses from six other geographical origins collected during the same seasons in Greece, the respective values for the discrimination of geographical origin of all eleven origins were 89.3% for conventional quality parameters plus minerals; 94.0% for conventional quality parameters plus fatty acids; 94.1% for minerals plus fatty acids; and 95.2% for conventional quality parameters plus minerals plus fatty acids. Such high correct classification rates demonstrate the robustness of the developed statistical model.

## 1. Introduction

“Graviera” is a gruyère-type hard, salty table cheese with a pleasant aroma and fine taste. Its texture is firm, having a smear rind and usually exhibiting small or larger irregular eyes. It is of light yellow color and is produced in various regions in Greece as a protected designation of origin (PDO) and non-PDO product. It is traded under the name of the region where it is produced (graviera of Crete, graviera of Naxos, graviera of Amfilochia, etc.).

It is the most popular hard Greek cheese and is second only to feta in consumption. It has a minimum fat content of 40% (dry weight basis—dwb) and a maximum moisture content of 38% [1].

In regular practice, graviera is produced from a mixture of 80% sheep milk with the addition of up to 20% of goat milk. However, graviera cheeses made of 100% sheep, goat, or cow milks, or a mixture of all three kinds of milk can be found in various parts of Greece. For example, graviera of Crete is produced either from either 100% sheep milk or from 80% sheep milk and 20% goat milk, graviera of Ioannina is produced both from 100% cow milk or from 80% sheep milk and 20% goat milk, graviera of Amphilochia is produced from 100% sheep milk, graviera of Naxos island is produced from 80% cow milk and 20% goat milk, while graviera of Tinos island is produced from 100% cow milk [2]. It is quite clear that the composition and the sensory properties of different milk types, and consequently of cheeses, may vary substantially. Furthermore, factors such as the animal breed, agroclimatic conditions, season, type of feeding, time of milking, the flora of the local pasture, types of starter cultures used, as well as traditional cheese-making practices comprise sources of product variation [3,4].

As early as 1992, the EU has recognized and supported the potential of differentiating quality products on a regional basis by introducing the following geographical indications for a food product: protected designation of origin (PDO), protected geographical indication (PGI), and traditional specialties guarantee (TSG) [1,5]. Such authentic products with unique compositions or processing characteristics enjoy higher prices on domestic and international markets. Of all graviera cheeses produced in Greece, only “graveria of Crete”, “graveria of Naxos”, and “graveria of Agrafa” are registered within the EU scheme as PDO products [6].

Chromatographic, spectroscopic, and molecular techniques have been used to determine the authenticity of dairy products, including gas chromatography (GC), gas chromatography/mass spectrometry (GC/MS) [7], high-performance liquid chromatography (HPLC) [8], liquid chromatography/mass spectrometry (LC/MS) [9,10], Fourier-transform infrared spectrometry (FTIR) [11], near-infrared (NIR) spectrometry [12], inductively coupled plasma spectrometry (ICP) [13], isotope ratio mass spectrometry (IRMS) [14], nuclear magnetic resonance (NMR) spectrometry [15,16], and molecular techniques [17].

Numerous studies on the geographical authentication of cheeses have been published in the literature. Curioni and Bosset [18] used GC–olafctometry to determine volatile compounds, including terpenes, to discriminate cheeses according to their production area. Pillonel et al. [19] used different FTIR spectroscopic techniques in combination with multivariate chemometrics to investigate their potential for discriminating 20 emmental cheeses from six different geographical origins. Brescia et al. [20] combined different techniques (high-performance ion chromatography, inductively coupled plasma–atomic emission spectroscopy, nuclear magnetic resonance, and isotope ratio mass spectrometry), together with chemometric methods to determine different compounds, enabling the geographical characterization of buffalo milk mozzarella cheese originating from two areas of southern Italy. Suhaj and Korenovska [21] used atomic absorption spectrometry in combination with cluster, principal component, factor, and discriminant analyses to trace the geographical origin of some emmental and edam hard cheeses originating from ten European countries. Rodríguez et al. [22] used high-performance liquid chromatography with diode-array detection (HPLC-DAD) to determine the protein profiles of cheeses, and consequently also of the types of milk used. Bozoudi et al. [23] investigated the microbiological and physicochemical characteristics as well as volatile profiles of graviera of Naxos and graviera of Crete, in order to differentiate the origins of these two types of cheeses. Bontempo et al. [24] used stable isotope ratio analysis and elemental analysis in combination with chemometrics to determine the authenticity of Italian PDO cheeses. Finally, Danezis et al. [25] used ICP-MS to determine a comprehensive elemental profile of Greek graveria cheeses in an effort to differentiate the cheeses by geographical origin and milk type.

The present study comprises the second half of the work recently published [26] by our group, with the main objective of characterizing and discriminating selected Greek graviera cheeses according to geographical origin, which were produced in five different regions of Greece (Ioannina, Trikala, Lesvos, Tinos, and Chania-Crete). A second objective, which was considered a challenge given the large total number of cheeses originating from 11 different geographical areas of Greece (six in our previous study and five in the present one), was to attempt to discriminate all eleven cheese geographical origins using the same developed statistical model.

Among the cheeses analyzed, “graveria of Tinos” was included, which as stated above is produced from 100% cow milk, thus, anticipating its clear differentiation from all other graviera cheese samples produced from 80% sheep milk and 20% of goat milk.

## 2. Results and Discussion

### 2.1. Determination of Conventional Quality Parameters (CQPs)

Table 1 depicts graviera cheese CQP. The pH values ranged between 5.58 ± 0.02 for graviera from Trikala and 5.73 ± 0.13 for graviera from Ioannina. Statistically significant differences (*p* < 0.05) in pH were recorded for these two cheeses. TA values varied between 0.61% ± 0.05 for graviera from Ioannina and 0.92% ± 0.13 lactic acid for graviera from Tinos. Statistically significant differences (*p* < 0.05) in TA were recorded between graviera from Tinos and all other graviera cheeses. Large differences (*p* < 0.05) were recorded in the NaCl contents, which ranged from 1.82 ± 0.07 for graviera from Chania to 3.80 ± 0.28 g/L for graviera from Ioannina. Moisture varied between 31.59% ± 1.28 for graviera from Chania and 36.67% ± 2.17 for graviera from Ioannina, with statistically significant differences (*p* < 0.05) recorded between graviera from Chania and all other graviera cheeses. Similarly to NaCl, graviera cheeses showed large differences (*p* < 0.05) in ash content. Ash ranged between 3.86% ± 0.64 for graviera from Lesvos and 6.29% ± 0.50 for graviera from Ioannina. The percentages of fat dry weight basis (dwb) varied between 45.97% ± 3.53 for graviera from Tinos and 51.57% ± 3.75 for graviera from Lesvos. Graviera cheeses from Ioannina and Lesvos recorded higher % fat dwb (*p* < 0.05) than all other cheeses. Finally, protein percentages ranged between 25.20% ± 1.14 for graviera from Ioannina and 30.56% ± 2.13 for graviera from Chania. Graviera cheeses from Tinos and Chania recorded higher % protein (*p* < 0.05) than all other cheeses.

Zerfiridis et al. [27] reported on the pH, moisture content, fat dwb, and salt values for Greek graviera cheese ripened for three months, equaling 5.76, 38.08%, 54.57%, and 1.31%, respectively. The above values compare to those in the present study as follows—they were similar in pH and moisture content, lower in fat dwb content, and higher in salt content (with the exception of graviera from Chania). Samelis et al. [28] reported pH values for graviera cheese of between 5.4 and 5.8 and a salt content of 1.6%. Differences in salt contents between the above values and those of the present study are attributed to different local cheese making practices. Finally, Vatavali et al. [26] analyzed graviera cheeses produced in Greece (Etoloakarnania, Arta, Thessaloniki, Messinia, Naxos, and Rethymno) and reported similar pH, protein, fat dwb, and moisture content values; similar NaCl values (with the exception of graviera from Chania showing a lower NaCl content); similar TA values (with the exception of graviera from Tinos showing higher TA values); and similar ash values (with the exception of graviera from Ioannina showing higher ash values). Apparently, a higher cheese NaCl content is accompanied by a higher ash content.

### 2.2. Determination of Mineral Content

Mineral content may be mainly related to milk composition, which in turn is related to milk origin (cow, sheep, goat, buffalo, etc.) and geographical origin. Table 2 shows a total of 24 minerals quantified in graviera cheese samples. The total amounts of minerals in decreasing order were: Lesvos (13,621.79 mg/kg) > Ioannina (8933.80 mg/kg) > Tinos (8103.66 mg/kg) ≈ Trikala (8086.95 mg/kg) > Chania (6754.21 mg/kg).

The most abundant minerals determined were the macro-elements: Na, Ca, and P followed by micro-elements: Mg, Zn, Sr, Fe, etc. In most cases there were statistically significant differences (*p* < 0.05) in mineral concentration among cheeses from different geographical origin. Results of the present study regarding mineral content are in general agreement with those of Gonzalez-Martin et al. [29] who determined the mineral content of cow, sheep and goat milk from Zamora, Spain. Danezis et al. [25] reported the signature of 61 elements in Greek graviera cheeses including all sixteen rare earth elements and all seven precious metals which were used to differentiate cheese geographical origin. Likewise, Vatavali et al. [26] determined among other analytical parameters, a similar profile of minerals for graviera cheeses from six different geographical origins in Greece. Total mineral content ranged between 16,489.46 mg/kg (for Messinia graviera samples) and 6490.04 mg/kg (for Rethymno cheese samples) with Ca, P, Na, Mg and Zn being the most abundant minerals.

### 2.3. Semi-Quantitative Determination of Volatile Compounds (VCs)

Proteolysis, lipolysis, and glycolysis are the main ripening processes involved in the formation of cheese volatiles. Among these biochemical processes, proteolysis is the most important for the formation of flavor and texture in hard-type and semi-hard-type cheeses [30,31]. The water soluble fraction (WSF) of ripened cheeses has been investigated extensively, as it has been documented to greatly contribute to cheese flavor [32]. WSF contains numerous non-volatile and volatile compounds resulting from the breakdown of amino acids, fat, lactose, and citrate during ripening [33].

Table 3 depicts graviera cheese volatiles originating from five different regions in Greece. A total of 57 compounds were identified in cheeses from all five regions, in the following descending order: carboxylic acids > ketones > alcohols > esters > hydrocarbons > miscellaneous > aldehydes.

Carboxylic acids varied between 0.782 mg/kg for the graviera from Lesvos and 4.145 mg/kg for the graviera from Chania. Ketones varied between 0.246 mg/kg for the graviera from Tinos and 2.017 mg/kg for the graviera from Chania. Alcohols varied between 0.073 mg/kg for the graviera from Trikala and 1.204 mg/kg for the graviera from Chania. Esters ranged from 0.044 mg/kg for the graviera from Trikala and 1.088 mg/kg for the graviera from Chania. Hydrocarbons varied between 0.042 mg/kg for the graviera from Tinos and 0.164 mg/kg for the graviera from Trikala. Miscellaneous volatiles varied between 0.014 mg/kg for the graviera from Tinos and 0.126 mg/kg for the graviera from Chania. Finally, aldehydes were present in minute amounts ranging from 0.011 mg/kg for the graviera from Tinos to 0.051 mg/kg for the graviera from Chania. A repetitive trend observed is that graviera from Tinos showed the lowest amounts in almost all volatile classes, attributed to the fact that this graviera cheese is produced from 100% cow milk, which contains less fat and protein than goat or sheep milk [34]. In most cases statistically significant differences (*p* < 0.05) in specific volatiles’ concentration were recorded among cheeses of different geographical origin.

Data in Table 3 show that the graviera from Chania had the highest VC content (8.754 mg/kg) among the cheeses of the five geographical origins. The amounts of cheese volatiles in descending order were: Chania >> Ioannina ≈ Lesvos > Trikala > Tinos. The milk used for graveria production comes from breeds that are raised locally, which feed on a diet of local plants and herbs that give their milk a unique flavor, in part due to VCs. Given that all dairies used the same starter cultures, we assume that the specific VCs determined were primarily dependent on animal diets and were secondarily dependent on animal breed.

Engels et al. [33] investigated the water soluble fraction (WSF) of gruyere cheeses and reported six major groups of VC compounds, namely fatty acids, esters, aldehydes, alcohols, ketones, and sulfur compounds. Likewise, Mallia et al. [35] investigated the volatile fraction of Swiss gruyere cheeses produced from raw milk and reported high concentrations of alkenes, aldehydes, methyl ketones, butane-2,3-dione, unsaturated alcohols, branched chain acids, and 2,6-dimethyl pyrazine for Swiss gruyere cheeses. Finally, Vatavali et al. [26] identified and semi-quantified a total of 61 compounds in graviera cheeses from six different regions in Greece, which in descending order were carboxylic acids (14.610 mg/kg), esters (13.854 mg/kg), alcohols (4.061 mg/kg), hydrocarbons (1.989 mg/kg), miscellaneous (1.075 mg/kg), ketones (0.628 mg/kg), ethers (0.048 mg/kg), and aldehydes (0.032 mg/kg), in general agreement with the results of the present study.

### 2.4. Determination of Fatty Acid (FA) Composition

Table 4 depicts the FA compositions of graviera cheeses from five different geographical regions. The fatty acids quantified in graviera samples included butyric acid (C_4:0_), caproic acid (C_6:0_), caprylic acid (C_8:0_), capric acid (C_10:0_), lauric acid (C_12:0_), myristic acid (C_14:0_), pentadecanoic acid (C_15:0_), palmitic acid (C_16:0_), palmitoleic acid (C_16:1_), margaric acid (C_17:0_), stearic acid (C_18:0_), oleic acid (C_18:1_), linoleic acid (C_18:2_), linolenic acid (C_18:3_), and arachidic acid (C_20:0_). The dominant FAs in terms of concentrations for graviera cheeses were palmitic acid, oleic acid, stearic acid, myristic acid, capric acid, and butyric acid. Palmitic acid was significantly different (*p* < 0.05) from most cheese samples, with its highest concentration (28.24% ± 0.37) recorded in the graviera from Tinos and its lowest concentration (22.70% ± 2.10) recorded in the graviera from Trikala. Oleic acid showed no statistically significant differences among graviera cheeses of different origins (with the exception of the graviera from Trikala and Tinos). Its highest concentration (27.79% ± 2.06) was recorded in the graviera from Trikala and its lowest concentration (21.70% ± 1.50) in the graviera from Chania. Stearic acid recorded its highest concentration (15.04% ± 0.78) in the graviera from Tinos and its lowest concentration (9.58% ± 0.91) in the graviera from Chania, being statistically different (*p* < 0.05) for most graviera cheeses of different origins. The myristic acid concentrations varied between 9.22% ± 0.82 (graviera from Trikala) and 11.69% ± 0.50 (graviera from Chania), being significantly different (*p* < 0.05) for most cheeses of different origins. Capric acid ranged between 2.53% ± 0.14 for the graviera from Tinos and 8.29% ± 0.94 for the graviera from Chania, being significantly different (*p* < 0.05) for most cheeses of different origins. The much lower capric acid content of graviera from Tinos compared to that of the other graviera cheeses may be attributed to the significantly lower capric acid content of the cow milk used to produce the graviera from Tinos compared to that of sheep milk, which is mainly used to produce all other graviera cheeses [34]. It should be noted that the concentration of fat in milk is dependent on the animal breed, nutrition, individual traits, and period of lactation. The profiles of FAs from goat, sheep, and cow milk fat differ [36]. The fat from sheep and goat milks are characterized by higher levels of medium-chain FAs (C6:0, C8:0 and C10:0) than those found in cow milk fat; these FAs are responsible for the typical aroma of the milk from small ruminants [37]. Finally, butyric acid varied between 4.30% ± 1.97 (graviera from Chania) and 4.78% ± 0.50 (graviera from Ioannina), showing no statistically significant differences among graviera cheeses of different geographical origins.

Zerfiridis et al. [27] reported the dominant FAs in commercial Greek graviera cheeses in descending order to be palmitic, myristic, linoleic and linolenic, lauric, capric, caprylic, and oleic acids. Zlatanos and Laskaridis [38] determined the FA composition of graviera cheeses and reported the major FAs in descending order to be palmitic acid (22.38%), oleic (20.84%), myristic (10.48%), capric (9.58%), and stearic (9.52%) acids. These researchers concluded that the differences in FA concentrations should be adequate to differentiate graviera cheeses on the basis of FA content. Vatavali et al. [26] reported similar values for the same 15 FAs quantified, which originated from six different areas in Greece. The dominant FAs in order of decreasing concentration were palmitic, oleic, stearic, myristic, and capric acids, in agreement with findings of the present study.

### 2.5. Discrimination of Graviera Cheeses Based on CQP

**Figure 1:**
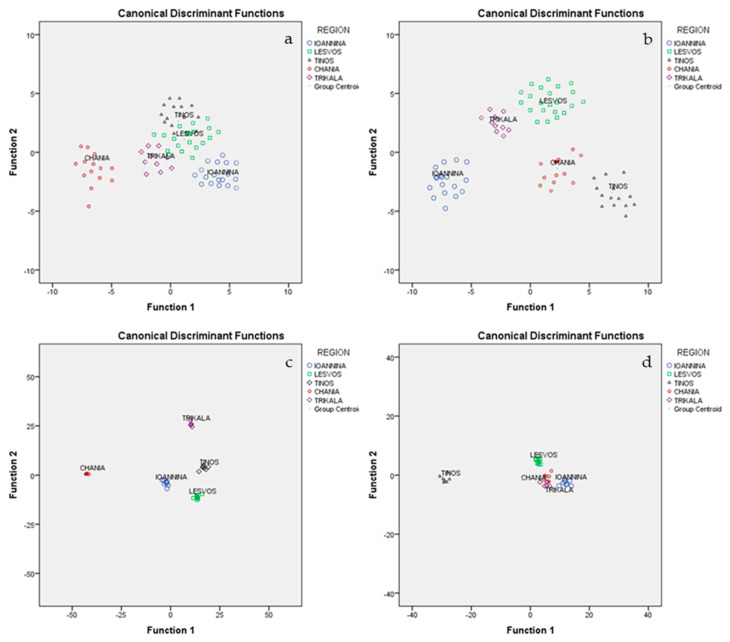
Geographical differentiation of graveria cheeses based on (**a**) CQPs, (**b**) minerals, (**c**) VCs, and (**d**) FA compositions.

All 78 graviera cheese samples were subjected to MANOVA in order to determine those CQP, that are significant for the geographical discrimination of graviera cheeses. Measured CQPs were taken as the dependent variables, while geographical origin was taken as the independent variable. Pillai’s trace = 2.687 (*F* = 9.068, *p* = 0.001 < 0.05) and Wilks’ lambda = 0.004 (*F* = 13.725, *p* = 0.001 < 0.05) index values showed the existence of a significant multivariable effect of CQPs on geographical origin. All 7 CQPs were found to be significant (*p* < 0.05) for the discrimination of cheese geographical origin and were, thus, subjected to LDA. The results showed that of the three statistically significant discriminant functions formed, the first discriminant function accounted for 71.5% of the total variance (canonical *R*^2^ = 0.966), the second accounted for 17.3% (canonical *R*^2^ = 0.880) and the third accounted for 8.4% (canonical *R*^2^ = 0.790)([Wilks’ Lambda = 0.004, *X*^2^ = 179.869, *df* = 28, *p* = 0.001 < 0.05 (1st); Wilks’ Lambda = 0.055, *X*^2^ = 92.971, *df* = 18, *p* = 0.001 < 0.05 (2nd); and Wilks’ Lambda = 0.242, *X*^2^ = 45.380, *df* = 10, *p* = 0.002 < 0.05 (3rd)). All three accounted for 97.2%, a very satisfactory value. Testing of the uniformity of variability (Box M index = 202.137, F = 1.428, *p* = 0.070 > 0.05) was insignificant at the 95% confidence level, showing the existence of uniformity of sample variability for each geographical origin. These three discriminant functions significantly discriminated the geographical origins of graviera cheeses. The highest classification rate (100%) was achieved for Chania, followed by Tinos (85.7%) and Trikala (80%), while the other regions achieved lower classification rates. The overall correct classification rate was 100% for the original method and 82.6% for the cross-validation method (Figure 1a). Vatavali et al. [26] determined the conventional quality parameters (pH, titratable acidity, moisture, fat, protein, ash, and NaCl) of 90 graviera cheeses from six different geographical origins and reported a low correct classification rate of 64.3% using the cross-validation method.

### 2.6. Discrimination of Graviera Cheeses Based on Minerals

Following the application of MANOVA analysis to the 24 minerals determined for the 78 graviera cheeses, it was found that 15 minerals were significant (*p* < 0.05) for the discrimination of cheese geographical origin and were, thus, subjected to LDA. Of the three statistically significant discriminant functions formed, the first discriminant function accounted for 62% of the total variance, the second accounted for 22.1%, and the third accounted for 10.3%. All three accounted for 94.4%, a very satisfactory value. The above discriminant functions led to a classification rate of 100% for Tinos and Ioannina, followed by 85.7% for Chania, 80% for Trikala, and 81.8% for Lesvos. The overall correct classification rate was 100% for the original method and 89.8% for the cross-validation method (Figure 1b).

Suhaj and Korenovska [21] determined the elemental markers Ba, Ca, Cr, Cu, Hg, K, Mg, Mn, Mo, Na, Ni, and V in an effort to trace the geographical origin of emmental and edam hard cheeses originating from ten European countries. The application of cluster analyses to experimental data indicated partial grouping of cheeses according to the country of origin. The application of canonical discriminant analysis to data resulted in percentages of correct classification and prediction in the range of 85.9–93% based on multi-elemental data. Osorio et al. [39] analyzed samples of milk and halloumi cheese for major and trace elements using inductively coupled plasma–atomic emission spectroscopy (ICP-AES). Samples originated from three different locations in Cyprus. Among the elements determined, Ag, Ba, K, Mn, and Sr showed potential for the geographical differentiation of halloumi cheeses. The application of canonical discriminant analysis considering the above five elements showed a correct classification of 95.7% using the cross-validation method. Camin et al. [40] developed two statistical models, the first based on isotopic composition and the second on elemental composition, in order to determine the origins of cheeses in grated and shredded forms. The first model was successful in predicting the origins of seven types of European hard cheeses. while the second specifically discriminated the PDO parmigiano reggiano cheese from 9 European and 2 non-European imitators. The most significant variables for cheese traceability common in both models were *δ*^13^C, *δ*^2^H, *δ*^15^N, *δ*^34^S and Sr, Cu, Mo, Re, Na, U, Bi, Ni, Fe, Mn, Ga, Se, and Li. Likewise, Bontempo et al. [24] used stable isotope ratio analysis along with elemental analysis to detect the authenticity of Italian PDO cheeses. The combined isotopic and mineral parameters proved useful for the discrimination between milk and cheese from the four different geographical areas (Salerno, Caserta, Foggia, and Latina), all of which are in the south of Italy. The classification rate was 95.8% for milk and 98.5% for mozzarella cheese. However, the method was not always able to discriminate between PDO and non-PDO buffalo mozzarella originating from the same production area. Recently, Danezis et al. [25] determined the elemental profile of 105 graviera cheeses from nine different geographic regions in Greece in an effort to differentiate the cheeses based on geographical origin and milk type. Cheeses were produced from sheep, goat, and cow milk, as well as their mixtures. Application of LDA to the experimental data gave a correct classification rate of 91.9 5 for milk type, while the differentiation of graviera cheese according to geographical origin was less successful. Finally, Vatavali et al. [26] determined the mineral content of 90 graviera cheeses from six different geographical origins and reported a high correct classification rate of 91.1% using the cross-validation method.

### 2.7. Discrimination of Graviera Cheeses Based on VCs

Following the application of MANOVA analysis to the 57 volatiles determined for the 78 graviera cheeses, it was found that 37 volatiles were significant (*p* < 0.05) for the discrimination of cheese geographical origin and were, thus, subjected to LDA. Of the two statistically significant discriminant functions formed, the first discriminant function accounted for 90.2% of total variance and the second accounted for 5%. Together they accounted for 95.2%, a very satisfactory value. The above discriminant functions led to a classification rate of 100% for Tinos, followed by 88.9% for Ioannina, 80% for Trikala, and 72.7% for Lesvos. The overall correct classification rate was 100% for the original method and 76.9% for the cross validation method (Figure 1c).

Bozoudi et al. [23] determined, among other parameters, the volatile profiles of the two cheeses (graviera of Naxos and graviera of Crete) in an effort to differentiate them. Results showed that the two cheeses had their own volatile profiles. Acids, esters, and alcohols were the most abundant groups of volatiles, in agreement with the volatiles determined in the present study. There were compounds common for both cheese types and others that were discriminant for each area. Vatavali et al. [26] determined the volatile profiles of 90 graviera cheeses from six different geographical origins and reported a very low correct classification rate of 46.7% using the cross-validation method.

### 2.8. Discrimination of Graviera Cheeses Based on FAs

Following the application of MANOVA analysis to the 15 FAs determined for the 78 graviera cheeses, it was found that 14 FAs were significant (*p* < 0.05) for the discrimination of cheese geographical origin and were, thus, subjected to LDA. Of the two statistically significant discriminant functions formed, the first discriminant function accounted for 91.7% of the total variance, while the second accounted for 4.7%. Both together accounted for 96.3%, a very satisfactory value. The above discriminant functions led to a classification rate of 100% for Tinos and Lesvos, followed by 88.9% for Ioannina, 80% for Trikala, and 71.4% for Chania. The overall correct classification rate was 100% for the original method and 89.7% for the cross-validation method (Figure 1d).

Vargas-Bello-Perez et al. [16] were able to discriminate retail cheeses according to variety (gouda, chanco, and mantecoso) by MANOVA and PCA using FA profile differences. Gouda and chanco cheeses were differentiated by saturated FA (C6:0, C8:0, C10:0, C11:0, C12:0, C14:0, C16:0, and C18:0), whereas mantecoso cheese was differentiated by specific (C4:0, C14:1, C16:1, C17:0, and C18:1) fatty acids. Vatavali et al. [26] determined the fatty acid compositions of 90 graviera cheeses from six different geographical origins and reported a high correct classification rate of 91.1% using the cross-validation method.

In order to further increase the correct classification rate, various combinations of analytical data sets were tested.

### 2.9. Discrimination of Graviera Cheeses Based on the Combination of FA Compositions and Minerals

**Figure 2:**
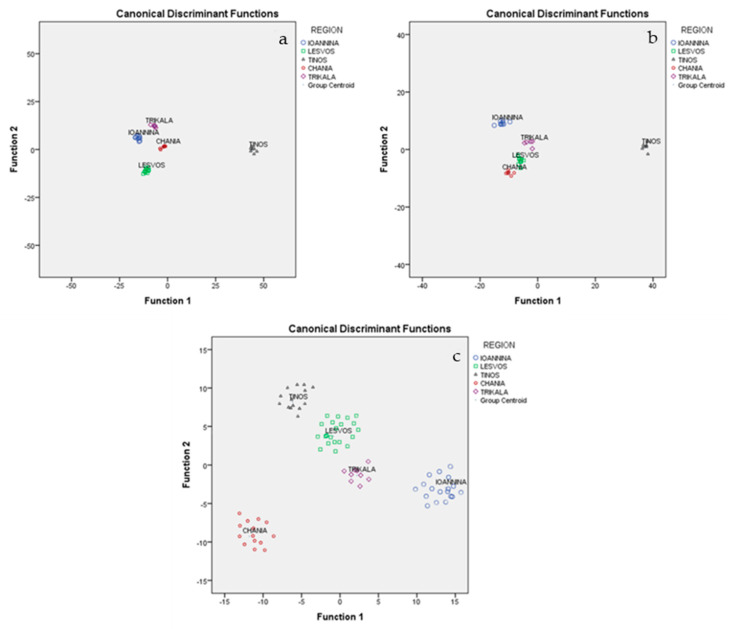
Geographical differentiation of graviera cheese based on the combinations of (**a**) FA composition and minerals, (**b**) CQP and FA composition and (**c**) CQP and minerals.

Following the application of MANOVA analysis to the FAs and minerals determined for the 78 graviera cheeses, it was found that 14 FAs and 15 minerals were significant (*p* < 0.05) for the differentiation of cheese geographical origin, and were, thus, subjected to LDA. Of the two statistically significant discriminant functions formed, the first discriminant function accounted for 81.4% of total variance and the second accounted for 11.2%. Both accounted for 92.6% of total variance, which is a very satisfactory value. Above discriminant functions led to a classification rate of 100% for Tinos, Lesvos, and Trikala, while the respective rate for the other regions were 77.8% for Ioannina and 71.4% for Chania. The overall correct classification rate was 100% for the original method and 89.7% for the cross-validation method (Figure 2a).

### 2.10. Discrimination of Graviera Cheeses Based on the Combination of FA Compositions and CQPs

Following the application of MANOVA analysis to the FAs and CQPs determined for the 78 graviera cheeses, it was found that 14 FAs and 7 CQPs were significant (*p* < 0.05) for the discrimination of cheese geographical origin, and were, thus, subjected to LDA. Of the two statistically significant discriminant functions formed, the first discriminant function accounted for 83.1% of the total variance and the second accounted for 9.6%. Both together accounted for 92.7% of the total variance, which is a very satisfactory value. The above discriminant functions led to a classification rate of 100% for Tinos, Lesvos, and Ioannina, while the respective rates for the other regions were 85.7% for Chania and 80% for Trikala. The overall correct classification rate was 100% for the original method and 94.9% for the cross-validation method (Figure 2b).

### 2.11. Discrimination of Graviera Cheeses Based on the Combination of Minerals and CQPs

Following the application of MANOVA analysis to the CQPs and minerals determined for the 78 graviera cheeses, it was found that 7 CQPs and 15 minerals were significant (*p* < 0.05) for the discrimination of cheese geographical origin, and were, thus, subjected to LDA. Of the three statistically significant discriminant function formed, the first discriminant function accounted for 59.2% of the total variance, the second accounted for 26%, and the third accounted for 12.3%. Together, these values accounted for 97.4% of the total variance, which is a very satisfactory value. The above discriminant functions led to a classification rate of 100% for all of the geographical regions, with the exception of Lesvos (90.9%). The overall correct classification rate was 100% for the original and 97.4% for the cross-validation method (Figure 2c).

### 2.12. Geographical Origin Discrimination of All Eleven Graviera Cheese Samples (Etoloakarnania, Arta, Thessaloniki, Messinia, Naxos, Rethymno, Ioannina, Trikala, Lesvos, Tinos, and Chania) Based on Analytical Parameters

In order to further test the performance of the developed model, we attempted to apply it to both the 168 presently and previously studied graviera cheese samples belonging to 11 different geographical origins.

Similarly, as described above, all 168 graviera samples were subjected to MANOVA in order to determine those CQPs, FAs, VCs, and minerals that are significant for the discrimination of geographical origin. In total, 54 of the 67 volatile compounds, 15 of 24 minerals, 14 of 15 FAs, and all 7 CQPs were found to be significant (*p* < 0.05) for the discrimination of cheese samples according to geographical origin, and were, thus, subjected to LDA. The overall correct classification rate was 69.0% for CQPs, 82.1% for minerals, 84.5% for FAs, and 64.3% for VCs using the cross-validation method, which were not very satisfactory rates regarding conventional quality parameters and volatiles, but were satisfactory rates regarding fatty acids and minerals.

**Figure 3:**
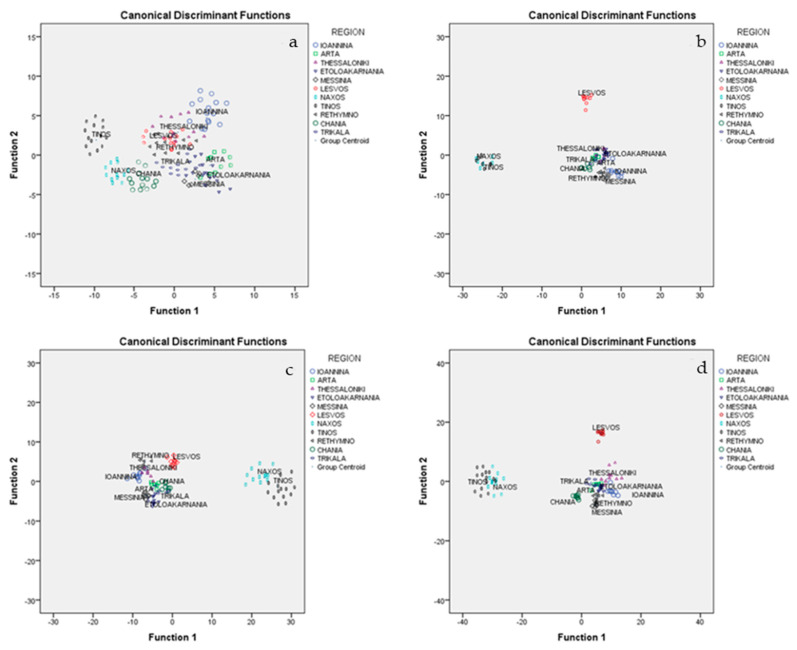
Geographical differentiation of graviera cheese from eleven regions based on the combinations of (**a**) minerals and conventional quality parameters (CQPs); (**b**) FA composition and minerals; (**c**) FA composition and CQPs; and (**d**) FA composition, minerals, and CQPs.

To further improve the above classification rates based on sets of individual analytical parameters, similar statistical treatment was used for combinations of analytical sets of data. The combination of CQPs, VCs, FAs, and minerals gave a respective correct classification rate of 72.6% using the cross-validation method; the combination of minerals and CQPs gave a correct classification rate of 89.3% (Figure 3a); the combination of FAs and minerals gave a correct classification rate of 94.0% (Figure 3b); the combination of FAs and CQPs gave a correct classification rate of 94.1% (Figure 3c); and finally the combination of FAs, minerals, and CQPs gave a correct classification rate of 95.2% (Figure 3d), which is considered an excellent rate considering the large number (11 in total) of geographical origins to be discriminated.

Based on the above results, it is clear that the developed statistical model performs extremely well for the discrimination of the geographical origin of a large number of graviera cheese samples.

## 3. Materials and Methods

### 3.1. Sample Collection

Seventy-eight samples of graviera cheese from 5 different regions in Greece (18 samples from Ioannina, 10 samples from Trikala, 22 samples from Lesvos, 14 samples from Tinos, and 14 samples from Chania) (Figure S1) were collected from specific small local dairies in Greece during the early spring period in 2017 (39 samples) and 2018 (39 samples).

### 3.2. Sample Preparation and Handling

Graviera cheeses were prepared as described by Vatavali et al. [26] in cooperating dairies following the same cheese-making procedure. All dairies used the same starter cultures; that is, a mixture of *Streptococcus thermophilus, Lactobacillus bulgaricus*, and *Lactobacillus helveticus*. An eye-forming starter of *Propionibacterium shermanii*, was also added (Chr. Hansen Holding A/S, Hørsholm, Denmark). Ninety-day-old ripe graviera samples were collected and shipped to the laboratory in polystyrene isothermal boxes in ice. Refrigerated samples were used for physicochemical and instrumental analyses. All determinations were carried out in triplicate.

### 3.3. Determination of CQP

The pH, TA, moisture, fat dwb, protein, NaCl, and ash values were determined using official methods of analysis [41].

### 3.4. Determination of Mineral Content Using Inductively Coupled Plasma–Optical Emission Spectroscopy (ICP-OES)

Cheese minerals were determined according to the method of Vatavali et al. [26]. In brief, a sample portion was mixed with aliquots of nitric acid 65% suprapur grade and 30% hydrogen peroxide, then the mixture was introduced into the microwave system for digestion. After cooling, the sample was analyzed using inductively coupled plasma–optical emission spectroscopy (ICP-OES) on an IRIS Intrepid II XDL instrument (Thermo Electron Corporation, Waltham, MA, USA). For the operational conditions used for the instruments, see Vatavali et al. [26]. All determinations were carried out in triplicate. Results were expressed as mg/kg.

### 3.5. Semi-Quantitative Determination of VCs Using Solid Phase Microextraction-Gas Chromatography/Mass Spectrometry (SPME-GC/MS)

Cheese volatiles were identified and semi-quantified according to the method of Vatavali et al. [26]. In brief, cheese samples along with an internal standard solution were placed in a glass serum vial and solid-phase microextraction (SPME) was performed with a DVB/CAR/PDMS 50/30 μm fiber (Supelco, Bellefonte, PA, USA). After equilibration, the fiber was inserted into the GC injection port and the analysis of VCs was carried out on a 60 m × 320 μm i.d. DB-5 ms column with a film thickness of 1 μm (J&W Scientific, Folsom, CA, USA). An Agilent Technologies 7890A GC system equipped with an Agilent Technologies 5975C MS system detector was used (Wilmington, DE, USA). Semi-quantification of VCs was carried out using the internal standard method. Concentrations were calculated using the following formula:

$$C_{x}=\frac{AREAx\cdot C_{I}}{AREA_{I}}$$

where *C_x_* = concentration of the unknown compound, *C_I_* = concentration of the internal standard solution, *AREAx* = peak area of the unknown compound, and *AREA_I_* = peak area of the internal standard solution. All determinations were carried out in triplicate. Results were expressed as mg/kg.

### 3.6. Determination of FA Composition Using Gas Chromatography/Flame Ionization Detector (GC/FID)

The FA composition was determined via transesterification into fatty acid methyl esters (FAME) according to the method of Gatzias et al. [7]. Briefly, the methyl esters were prepared by vigorous shaking of the lipid solution in heptane with a methanolic solution of KOH. After centrifugation, the supernatant layer containing the methyl esters was used for GC analysis. FAME samples were analyzed on a 6890N Agilent Technologies GC-FID chromatograph (Wilmington, DE, USA), equipped with a 30 m × 320 μm i.d. Supelcowax column with a film thickness of 0.5 μm (Supelco, Bellefonte, USA). All determinations were carried out in triplicate. Results were expressed as % (*w/w*).

### 3.7. Statistical Analysis

Analytical data were treated using the SPSS 23.0 Statistics software [42]. Comparison of the means was achieved by MANOVA in order to determine those parameters that are significant in differentiating cheeses according to geographical origin. The independent variable was the geographical origin, while CQPs (pH, TA, moisture, protein, fat dwb, ash), FAs, VCs, and minerals were taken as the dependent variables. Pillai’s trace and Wilk’s lambda indices were computed in order to determine possible significant effects of conventional quality, chromatographic parameter, and element values on the geographical origins of graviera cheeses. LDA was then applied using the selected dependent variables to explore the possibility of classifying cheeses according to geographical origin. Both the original and leave-one-out cross validation methods were used to test the prediction ability. Cross validation is a more conservative method for correct classification, but at the same time is a more reliable one. In addition, the homogeneity of the variability was tested by application of the Box M index [43].

## 4. Conclusions

Analysis of conventional quality parameters, minerals, volatile compounds, and fatty acids showed significant differences among graviera cheeses produced in different geographical regions in Greece. Statistical treatment of the individual sets of data gave acceptable but not satisfactory correct classification rates according to geographical origin (conventional quality parameters: 82.6%, minerals: 82.1%, volatile compounds: 64.3%, and fatty acids: 84.5%). Furthermore, combinations of selected analytical data sets increased the correct classification rate (i.e., fatty acids and minerals: 89.7%, fatty acids and conventional quality parameters: 94.9%, and minerals and conventional quality parameters: 97.4%). Finally, in order to test the performance of the developed statistical model, an attempt was made to discriminate graviera chesses collected from (i) five different geographical regions of the present study and (ii) six different regions in Greece from our previous study. Discrimination of all eleven geographical regions was achieved with a classification rate of 89.3% for the combination of conventional quality parameters and minerals; 94.0% for the combination of minerals and fatty acids; 94.1% for the combination of fatty acids and conventional quality parameters; and 95.2% for the combination of fatty acids, minerals, and conventional quality parameters. The above classification rate values show the excellent performance of the statistical model developed for the discrimination of the geographical origin of a large number of graviera cheeses.

## Figures and Tables

**Table 1:** Mean values and standard deviations (SD) of conventional quality parameters for the graviera cheese samples tested.

	*Ioannina*	*Lesvos*	*Tinos*	*Chania*	*Trikala*
pH	5.73 ± 0.13 ^b^	5.62 ± 0.05 ^a,b^	5.67 ± 0.04 ^a,b^	5.68 ± 0.08 ^a,b^	5.58 ± 0.02 ^a^
NaCl (g/L)	3.80 ± 0.28 ^d^	3.05 ± 0.52 ^b,c^	3.55 ± 0.27 ^c,d^	1.82 ± 0.07 ^a^	2.66 ± 0.18 ^b^
Titratable Acidity (lactic acid%)	0.61 ± 0.05 ^a^	0.72 ± 0.08 ^a^	0.92 ± 0.13 ^b^	0.67 ± 0.12 ^a^	0.65 ± 0.10 ^a^
Moisture (%)	36.67 ± 2.17 ^b^	36.27 ± 1.10 ^b^	36.70 ± 1.75 ^b^	31.59 ± 1.28 ^a^	35.33 ± 2.17 ^b^
Ash (%)	6.29 ± 0.50 ^d^	3.86 ± 0.64 ^a^	4.73 ± 0.45 ^a,b^	5.08 ± 1.14 ^c^	4.88 ± 0.56 ^a,b^
Fat dwb (%)	51.42 ± 1.67 ^b^	51.57 ± 3.75 ^b^	45.97 ± 3.53 ^a^	46.37 ± 1.62 ^a^	46.97 ± 2.07 ^a^
Protein (%)	25.20 ± 1.14 ^a^	26.20 ± 1.57 ^a^	29.35 ± 0.99 ^b^	30.56 ± 2.13 ^b^	26.08 ± 0.88 ^a^

Note: ^a^, ^b^, ^c^, ^d^ mean values with different superscripts in the same row are significantly different (Duncan’s test, *p* < 0.05).

**Table 2:** Mean values (mg/kg) and SDs of minerals for the graviera cheese samples tested.

	*Ioannina*	*Lesvos*	*Tinos*	*Chania*	*Trikala*
Al	0.58 ± 0.52 ^a^	0.93 ± 0.54 ^a^	0.32 ± 0.49 ^a^	0.52 ± 0.61 ^a^	0.43 ± 0.55 ^a^
As	0.07 ± 0.00 ^a^	0.07 ± 0.01 ^a^	2.48 ± 4.12 ^b^	0.18 ± 0.32 ^a^	0.07 ± 0.01 ^a^
Ba	1.00 ± 0.20 ^a^	3.82 ± 2.30 ^b^	1.03 ± 0.20 ^a^	1.21 ± 0.53 ^a^	2.32 ± 0.23 ^a^
Be	0.05 ± 0.00 ^a^	0.05 ± 0.01 ^a^	0.05 ± 0.01 ^a^	0.05 ± 0.01 ^a^	0.05 ± 0.01 ^a^
Ca	2378.56 ± 341.56 ^a^	5643.82 ± 1677.95 ^b^	3050.43 ± 507.20 ^a^	3450.71 ± 360.28 ^a^	3038.00 ± 180.11 ^a^
Cd	0.04 ± 0.00 ^a^	0.04 ± 0.01 ^a^	0.04 ± 0.01 ^a^	0.04 ± 0.01 ^a^	0.04 ± 0.00 ^a^
Co	0.04 ± 0.02 ^a^	0.03 ± 0.00 ^a^	0.03 ± 0.00 ^a^	0.08 ± 0.14 ^a^	0.03 ± 0.00 ^a^
Cr	0.80 ± 0.42 ^b^	0.57 ± 0.25 ^b^	0.19 ± 0.17 ^a^	0.12 ± 0.01 ^a^	0.52 ± 0.26 ^b^
Cu	0.89 ± 0.48 ^a,b^	0.68 ± 0.27 ^b^	0.35 ± 0.06 ^a^	1.51 ± 0.51 ^c^	0.60 ± 0.10 ^a,b^
Fe	2.76 ± 1.09 ^a^	6.08 ± 12.79 ^a^	1.45 ± 0.53 ^a^	3.63 ± 0.34 ^a^	2.95 ± 0.37 ^a^
Hg	0.09 ± 0.18 ^a^	0.22 ± 0.30 ^a^	0.11 ± 0.14 ^a^	0.28 ± 0.10 ^a^	0.32 ± 0.04 ^a^
Mg	130.67 ± 17.94 ^a,b^	165.19 ± 48.10 ^b^	109.01 ± 9.96 ^a^	140.14 ± 28.12 ^a,b^	143.80 ± 9.96 ^a,b^
Mn	0.41 ^b^ ± 0.06 ^c^	0.54 ± 0.20 ^c^	0.11 ± 0.06 ^a^	0.41 ± 0.11 ^b,c^	0.29 ± 0.05 ^b^
Mo	0.19 ± 0.05 ^a^	0.21 ± 0.03 ^a,b^	0.23 ± 0.03 ^a,b^	0.24 ± 0.02 ^b^	0.22 ± 0.02 ^a,b^
Ni	0.09 ± 0.05 ^a,b^	0.05 ± 0.01 ^a^	0.09 ± 0.06 ^a,b^	0.05 ± 0.01 ^a^	0.14 ± 0.12 ^b^
P	2025.67 ± 564.54 ^a^	6137.27 ± 2810.81 ^b^	2241.71 ± 829.21 ^a^	1651.29 ± 987.48 ^a^	2089.20 ± 1047.77 ^a^
Pb	0.09 ± 0.06 ^a^	0.08 ± 0.01 ^a^	0.08 ± 0.01 ^a^	0.08 ± 0.01 ^a^	0.08 ± 0.01 ^a^
Sb	0.40 ± 0.24 ^a^	0.48 ± 0.16 ^a^	0.38 ± 0.09 ^a^	0.71 ± 0.11 ^b^	0.44 ± 0.09 ^a^
Se	0.13 ± 0.08 ^a^	0.29 ± 0.17 ^b^	0.11 ± 0.02 ^a^	0.11 ± 0.01 ^a^	0.11 ± 0.01 ^a^
Sr	10.14 ± 6.24 ^a^	17.77 ± 9.59 ^b^	7.57 ± 6.08 ^a^	23.67 ± 2.01 ^b,c^	25.93 ± 1.80 ^c^
Ti	0.41 ± 0.40 ^a^	0.46 ± 0.59 ^a^	0.99 ± 0.93 ^a,b^	0.60 ± 1.40 ^a,b^	2.31 ± 4.04 ^b^
Tl	0.98 ± 0.61 ^a^	1.08 ± 0.40 ^a^	1.02 ± 0.22 ^a^	1.75 ± 0.42 ^b^	2.06 ± 0.37 ^b^
Zn	21.75 ± 4.10 ^a^	42.42 ± 9.60 ^b^	42.29 ± 9.54 ^b^	52.27 ± 2.89 ^c^	35.82 ± 3.96 ^b^
Na	4358.00 ± 1776.77 ^b^	1599.64 ± 1232.58 ^a^	2643.57 ± 272.32 ^a^	1424.57 ± 877.20 ^a^	2741.20 ± 336.95 ^a^
Total	8933.80	13621.79	8103.66	6754.21	8086.95

Note: ^a^, ^b^, ^c^ mean values with different superscripts in the same row are significantly different (Duncan’s test, *p* < 0.05).

**Table 3:** Mean values (mg/kg) and SDs of volatile compounds (VCs) of the graviera cheese samples tested.

	*Ioannina*	*Lesvos*	*Tinos*	*Chania*	*Trikala*	RI exp *	RI lit **
*Alcohols*							
Ethanol	0.049 ± 0.059 ^a^	0.338 ± 0.308 ^a,b^	0.053 ± 0.045 ^a^	0.507 ± 0.382 ^b^	0.014 ± 0.009 ^a^	<500	<500
1-Propanol	0.001 ± 0.002 ^a^	0.003 ± 0.003 ^a^	0.000 ± 0.001 ^a^	0.017 ± 0.028 ^a^	-	552	554
2-Butanol	0.017 ± 0.043 ^a,b^	0.069 ± 0.045 ^b^	0.011 ± 0.023 ^a^	0.047 ± 0.042 ^a,b^	-	603	608
1-Butanol	-***	0.000 ± 0.001 ^a^	0.016 ± 0.017 ^b^	0.009 ± 0.005 ^a,b^	-	660	669
2-Pentanol	-	0.003 ± 0.004 ^a^	0.002 ± 0.004 ^a^	0.426 ± 0.269 ^b^	-	702	738
2,3-Butanediol	0.122 ± 0.048 ^c^	0.113 ± 0.029 ^c^	0.023 ± 0.023 ^a,b^	0.051 ± 0.027 ^b^	-	796	-
2-Methyl-3-pentanol	0.064 ± 0.048 ^b^	-	0.027 ± 0.026 ^a,b^	0.015 ± 0.040 ^a,b^	0.060 ± 0.025 ^b^	802	-
1-Hexanol	-	0.004 ± 0.006 ^a,b^	0.003 ± 0.005 ^a,b^	0.007 ± 0.005 ^b^	-	868	862
2-Heptanol	-	0.011 ± 0.024 ^a^	-	0.125 ± 0.072 ^b^	-	900	896
Total	0.253	0.540	0.135	1.204	0.073		
*Aldehydes*							
3-Methylbutanal	-	-	0.008 ± 0.009 ^a,b^	0.014 ± 0.008 ^b^	0.006 ± 0.010 ^a,b^	657	650
2-Methylbutanal	0.015 ± 0.018 ^a^	-	0.003 ± 0.008 ^a^	0.034 ± 0.054 ^a^	0.020 ± 0.044 ^a^	666	660
Heptanal	0.001 ± 0.001 ^a^	0.004 ± 0.009 ^a^	-	-	0.004 ± 0.004 ^a^	766	899
Nonanal	0.002 ± 0.002 ^a^	0.013 ± 0.035 ^a^	-	0.003 ± 0.002 ^a^	0.003 ± 0.003 ^a^	1108	1099
Total	0.018	0.017	0.011	0.051	0.032		
*Ketones*							
2-Propanone	0.022 ± 0.012 ^a,b^	0.011 ± 0.019 ^a^	0.012 ± 0.010 ^a^	0.052 ± 0.038 ^b^	0.018 ± 0.005 ^a^	<500	<500
2,3-Butanedione	0.009 ± 0.009 ^a^	0.007 ± 0.012 ^a^	0.009 ± 0.008 ^a^	0.009 ± 0.019	0.031 ± 0.009 ^b^	588	584
2-Butanone	0.149 ± 0.310 ^a^	0.153 ± 0.167 ^a^	0.017 ± 0.015 ^a^	0.103 ± 0.224 ^a^	0.006 ± 0.004 ^a^	595	600
2-Pentanone	0.013 ± 0.005 ^a^	0.023 ± 0.037 ^a^	0.014 ± 0.005 ^a^	0.911 ± 1.045 ^b^	0.013 ± 0.005 ^a^	686	684
3-Hydroxy-2-butanone	0.236 ± 0.164 ^a,b^	0.081 ± 0.045 ^a^	0.094 ± 0.066 ^a^	0.099 ± 0.227 ^a^	0.396 ± 0.092 ^b^	709	707
2-Heptanone	0.023 ± 0.011 ^a^	0.091 ± 0.236 ^a^	0.085 ± 0.059 ^a^	0.559 ± 0.254 ^b^	0.039 ± 0.027 ^a^	890	899
2-Nonanone	0.008 ± 0.004 ^a^	0.131 ± 0.392 ^a^	0.015 ± 0.010 ^a^	0.284 ± 0.290 ^a^	0.008 ± 0.005 ^a^	1092	1093
Total	0.459	0.496	0.246	2.017	0.512		
*Carboxylic Acids*							
Acetic acid	0.127 ± 0.091 ^a,b^	0.251 ± 0.056 ^a,b^	0.046 ± 0.062 ^a^	0.709 ± 0.941 ^b^	0.027 ± 0.032 ^a^	571	606
2-Methylpropanoic acid	0.006 ± 0.006 ^a,b^	0.035 ± 0.053 ^b^	-	-	-	737	753
Butanoic acid	0.753 ± 0.496 ^a^	0.282 ± 0.160 ^a^	0.544 ± 0.470 ^a^	1.529 ± 2.745 ^a^	0.659 ± 0.615 ^a^	774	784
Pentanoic acid	0.020 ± 0.016 ^a^	0.129 ± 0.197 ^a^	-	-	-	825	841
3-Methylbutanoic acid	0.015 ± 0.022 ^a^	0.000 ± 0.001 ^a^	0.020 ± 0.035 ^a^	0.008 ± 0.021 ^a^	-	825	842
2-Methylbutanoic acid	0.012 ± 0.008 ^a^	0.054 ± 0.080 ^a^	-	0.033 ± 0.086 ^a^	-	835	853
Hexanoic acid	0.525 ± 0.341 ^a^	0.030 ± 0.059 ^a^	0.008 ± 0.011 ^a^	1.574 ± 3.786 ^a^	0.362 ± 0.389 ^a^	966	970
Octanoic acid	0.059 ± 0.091 ^a^	-	-	0.293 ± 0.774 ^a^	0.035 ± 0.040 ^a^	1156	1177
Total	1.517	0.782	0.618	4.145	1.083		
*Esters*							
Ethyl acetate	0.005 ± 0.003 ^a^	0.015 ± 0.015 ^a^	0.002 ± 0.003 ^a^	0.055 ± 0.031 ^b^	0.011 ± 0.016 ^a^	611	614
Methyl butyrate	0.014 ± 0.005 ^a^	0.016 ± 0.007 ^a^	0.026 ± 0.013 ^a^	0.023 ± 0.014 ^a^	0.021 ± 0.010 ^a^	721	735
Ethyl butyrate	0.024 ± 0.018 ^a^	0.163 ± 0.188 ^a^	0.021 ± 0.019 ^a^	0.508 ± 0.308 ^b^	-	799	798
Ethyl pentanoate	-	0.002 ± 0.003 ^a^	-	0.002 ± 0.004 ^a^	-	898	901
Methyl hexanoate	0.010 ± 0.003 ^a^	0.018 ± 0.015 ^a^	0.012 ± 0.004 ^a^	0.025 ± 0.026 ^a^	0.009 ± 0.007 ^a^	923	934
Ethyl hexanoate	0.005 ± 0.011 ^a^	0.157 ± 0.291 ^a,b^	0.001 ± 0.004 ^a^	0.357 ± 0.210 ^b^	-	996	1001
Methyl octanoate	0.002 ± 0.002 ^a^	0.006 ± 0.009 ^a^	0.003 ± 0.002 ^a^	0.012 ± 0.017 ^a^	0.002 ± 0.003 ^a^	1122	1125
Ethyl octanoate	0.003 ± 0.004 ^a^	0.040 ± 0.088 ^a,b^	0.001 ± 0.001 ^a^	0.075 ± 0.054 ^b^	-	1193	1193
Methyl decanoate	0.001 ± 0.001 ^a^	0.004 ± 0.007 ^a^	0.000 ± 0.001 ^a^	0.003 ± 0.005 ^a^	-	1324	1326
Ethyl decanoate	0.001 ± 0.002 ^a^	0.028 ± 0.065 ^a^	-	0.029 ± 0.017 ^a^	-	1393	1391
Total	0.065	0.450	0.067	1.088	0.044		
*Hydrocarbons*							
2-Methylpentane	0.003 ± 0.005 ^a^	-	0.001 ± 0.002 ^a^	0.007 ± 0.010 ^a^	0.009 ± 0.013 ^a^	563	560
Cyclopentane	0.001 ± 0.002 ^a^	0.002 ± 0.003 ^a^	0.001 ± 0.001 ^a^	-	-	563	563
Hexane	0.014 ± 0.027 ^a^	0.001 ± 0.003 ^a^	0.013 ± 0.027 ^a^	0.003 ± 0.005 ^a^	0.013 ± 0.022 ^a^	600	600
Cyclohexane	0.006 ± 0.008 ^a^	0.000 ± 0.001 ^a^	-	0.022 ± 0.039 ^a^	0.020 ± 0.026 ^a^	668	658
Heptane	0.011 ± 0.010 ^a^	0.001 ± 0.002 ^a^	0.000 ± 0.001 ^a^	-	0.058 ± 0.049 ^b^	700	700
Octane	0.032 ± 0.036 ^a^	0.024 ± 0.023 ^a^	0.008 ± 0.010 ^a^	0.009 ± 0.009 ^a^	0.020 ± 0.044 ^a^	800	800
2-Octene	0.003 ± 0.003 ^a^	0.001 ± 0.002 ^a^	-	-	0.010 ± 0.007 ^b^	818	815
Ethylcyclohexane	-	0.003 ± 0.004 ^a,b^	-	-	0.005 ± 0.007 ^b^	845	838
Nonane	0.007 ± 0.002 ^b^	0.007 ± 0.005 ^b^	0.007 ± 0.001 ^b^	-	0.010 ± 0.005 ^b^	900	900
Decane	0.012 ± 0.002 ^a^	0.011 ± 0.003 ^a^	0.013 ± 0.004 ^a^	0.029 ± 0.016 ^b^	0.012 ± 0.004 ^a^	1000	1000
Undecane	-	0.005 ± 0.004 ^a^	-	0.030 ± 0.019 ^b^	0.006 ± 0.010 ^a^	1100	1100
Dodecane	-	-	-	0.014 ± 0.008 ^b^	0.003 ± 0.003 ^a^	1200	1200
Tridecane	-	-	-	0.011 ± 0.012 ^b^	-	1300	1300
Total	0.090	0.055	0.042	0.125	0.164		
*Miscellaneous*							
Ethyl ether	0.011 ± 0.011 ^a,b^	-	-	0.024 ± 0.034 ^a,b^	0.037 ± 0.054 ^b^	<500	<500
Dimethyl sulfide	0.002 ± 0.001 ^a,b^	0.001 ± 0.001 ^a,b^	0.003 ± 0.001 ^b^	0.001 ± 0.002 ^a^	0.006 ± 0.003 ^c^	519	526
α-Pinene	0.041 ± 0.026 ^b^	0.014 ± 0.011 ^a^	0.010 ± 0.002 ^a^	0.041 ± 0.025 ^b^	0.050 ± 0.012 ^b^	947	943
β-Pinene	0.007 ± 0.005 ^a^	0.008 ± 0.007 ^a^	-	0.002 ± 0.005 ^a,b^	0.005 ± 0.008 ^a,b^	994	978
o-Cymene	0.020 ± 0.024 ^a,b^	-	0.000 ± 0.001 ^a^	0.032 ± 0.033 ^b^	0.021 ± 0.020 ^a,b^	1037	1041
dl-Limonene	0.014 ± 0.010 ^a,b^	0.003 ± 0.010 ^a^	-	0.027 ± 0.016 ^b^	0.001 ± 0.001 ^a^	1042	1039
Total	0.095	0.027	0.014	0.126	0.120		
Total Volatile Fraction	2.497	2.368	1.132	8.754	2.029		

* RIexp: experimental retention index; ** RIlit: literature retention index (NIST MS search); *** **-** = not detected; ^a^, ^b^, ^c^ mean values with different superscripts in the same row are significantly different (Duncan’s test, *p* < 0.05).

**Table 4:** Mean values and SDs of the percentages of fatty acids (% fatty acids) for the graviera cheese samples tested.

	*Ioannina*	*Lesvos*	*Tinos*	*Chania*	*Trikala*
Butyric acid	4.78 ± 0.50 ^a^	4.41 ± 0.97 ^a^	4.57 ± 0.65 ^a^	4.30 ± 1.97 ^a^	4.76 ± 0.49 ^a^
Caproic acid	2.00 ± 0.29 ^a,b^	2.38 ± 0.11 ^c^	1.79 ± 0.09 ^a^	2.36 ± 0.20 ^c^	2.16 ± 0.24 ^b,c^
Caprylic acid	2.06 ± 0.36 ^b^	2.49 ± 0.22 ^c^	1.12 ± 0.06 ^a^	2.61 ± 0.24 ^c^	2.05 ± 0.30 ^b^
Capric acid	6.83 ± 1.13 ^b,c^	7.68 ± 1.12 ^c,d^	2.53 ± 0.14 ^a^	8.29 ± 0.94 ^d^	5.82 ± 0.79 ^b^
Lauric acid	3.97 ± 0.63 ^b,c^	4.62 ± 0.94 ^c,d^	2.91 ± 0.17 ^a^	4.97 ± 0.57 ^d^	3.40 ± 0.35 ^a,b^
Myristic acid	11.33 ± 0.27 ^b,c^	10.90 ± 1.47 ^b,c^	10.30 ± 0.49 ^a,b^	11.69 ± 0.50 ^c^	9.22 ± 0.82 ^a^
Pentadecanoic acid	1.21 ± 0.07 ^b^	0.92 ± 0.11 ^a^	1.12 ± 0.07 ^b^	1.09 ± 0.11 ^b^	1.21 ± 0.13 ^b^
Palmitic acid	27.63 ± 1.01 ^c,d^	24.62 ± 0.81 ^b^	28.24 ± 0.37 ^d^	26.63 ± 0.81 ^c^	22.70 ± 2.10 ^a^
Palmitoleic acid	1.20 ± 0.07 ^a,b^	1.12 ± 0.31 ^a^	1.46 ± 0.10 ^b^	1.20 ± 0.08 ^a,b^	1.33 ± 0.13 ^a,b^
Margaric acid	1.64 ± 0.49 ^b^	1.29 ± 0.21 ^a^	1.34 ± 0.21 ^a,b^	1.52 ± 0.17 ^a,b^	1.22 ± 0.18 ^a^
Stearic acid	10.50 ± 0.81 ^a^	11.67 ± 2.80 ^a,b^	15.04 ± 0.78 ^c^	9.58 ± 0.91 ^a^	13.31 ± 0.80 ^b,c^
Oleic acid	22.57 ± 1.30 ^a^	22.94 ± 1.78 ^a^	25.72 ± 0.80 ^a^	21.70 ± 1.50 ^a^	27.79 ± 2.06 ^b^
Linoleic acid	2.40 ± 0.22 ^a^	3.62 ± 0.51 ^b^	2.66 ± 0.28 ^a^	2.70 ± 0.15 ^a^	2.83 ± 0.32 ^a^
Linolenic acid	1.01 ± 0.11 ^b^	0.71 ± 0.08 ^a^	0.66 ± 0.07 ^a^	0.74 ± 0.41 ^a,b^	1.51 ± 0.07 ^c^
Arachidic acid	0.85 ± 0.21 ^b^	0.63 ± 0.12 ^a^	0.54 ± 0.05 ^a^	0.62 ± 0.15 ^a^	0.69 ± 0.07 ^a,b^

Note: ^a^, ^b^, ^c^, ^d^ mean values with different superscripts in the same row are significantly different (Duncan’s test, *p* < 0.05).

## Supplementary Materials

The following are available online at https://www.mdpi.com/1420-3049/25/15/3507/s1, Figure S1: A map of Greece showing the regions of graveria cheese samples.
