# Supplementary material for: Physicochemical, Spectroscopic, and Chromatographic Analyses in Combination with Chemometrics for the Discrimination of the Geographical Origin of Greek Graviera Cheeses
Source: Molecules. 2020 Jul 31;25(15):3507. doi: 10.3390/molecules25153507 (PMC7435398; doi:10.3390/molecules25153507)
Supplement: Supplementary file 1 [file molecules-25-03507-s001.pdf]

Supplementary

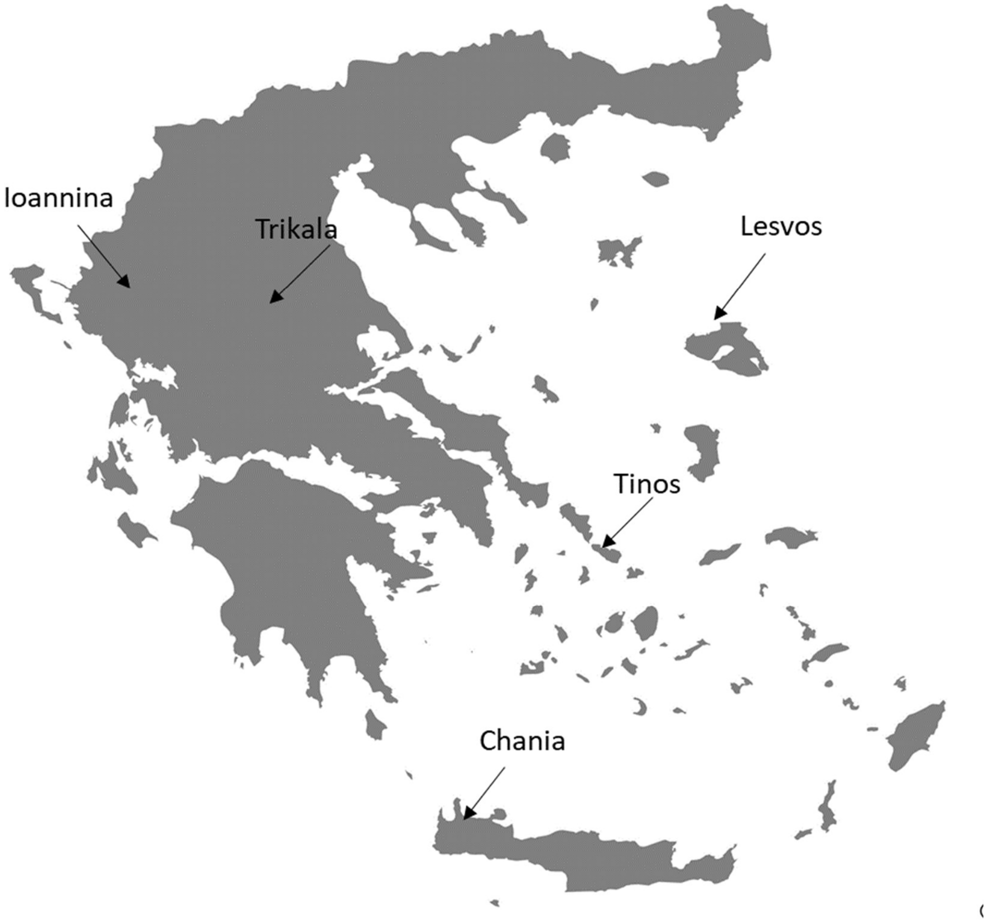

Figure S1. A map of Greece showing the regions of Graviera cheese samples collection.
